# Predictive modeling of sugar beet bolting via vernalization-intensity model and resilience assessment in diverse autumn cultivation environments

**DOI:** 10.1371/journal.pone.0339856

**Published:** 2026-02-26

**Authors:** Saeed Sadeghzadeh Hemayati, Ali Saremirad, Ahmad Nooshkam, Saeed Yar-Ahmadi, Moslem Abdipur

**Affiliations:** 1 Sugar Beet Seed Institute (SBSI), Agricultural Research, Education and Extension Organization (AREEO), Karaj, Iran; 2 Sugar Beet Research Department, Dezful Agricultural and Natural Resources Research and Education Center, AREEO, Dezful, Iran; 3 Sugar Beet Research Department, Golestan Agricultural and Natural Resources Research and Education Center, AREEO, Gonbad, Iran; 4 Seed & Plant Research Department, Chahar-Mahal Bakhtiary Agricultural and Natural Resources Research and Education Center, AREEO, Gachsaran, Iran; Assiut University, EGYPT

## Abstract

Climate change and increasing pressure on water resources have renewed interest in autumn cultivation of sugar beet, a practice that benefits from seasonal precipitation and reduces dependence on irrigation. However, bolting remains a major limitation, substantially affecting yield stability and the economic viability of production. This study evaluated ten experimental sugar beet hybrids and two bolting-resistant control varieties across three environments over two cropping years (2022–2023 and 2023–2024). Randomized complete block design with four replications was implemented for agronomic evaluations. The vernalization–intensity model was used to estimate vernalization threshold (VT) and bolting sensitivity. Genotype BOL436 exhibited the highest VT (134 h) but also showed pronounced sensitivity to bolting. In contrast, genotypes BOL435 and BOL434, with similarly high VT values, displayed low bolting sensitivity and thus greater suitability for fluctuating winter conditions. Combined ANOVA revealed significant genotype and environment effects (P < 0.01) for all measured traits, while genotype–environment interaction (GEI) significantly influenced white sugar yield (WSY) and root yield (RY). Additive main effects and multiplicative interactions (AMMI) analysis indicated that the first two interaction principal components (IPCs) accounted for 75.20% of GEI variation in WSY, whereas IPC1 alone explained 57.60% for RY. WAASB-based stability analysis identified BOL375, BOL239, BOL376, and BOL068 as stable, high-performing genotypes for WSY, with BOL239, BOL375, BOL068, and BOL435 showing comparable superiority for RY. Results from the WAASBY index were consistent with these findings. Multi-trait stability index (MTSI) analysis further highlighted BOL434, BOL376, and BOL239 as the most stable genotypes across agronomic and qualitative traits. Overall, the results demonstrate that autumn cultivation, supported by robust modeling and stability analysis, can contribute to reduced irrigation needs, while the identified genotypes offer promising options for enhancing productivity and minimizing bolting risk in water-limited environments.

## Introduction

Water scarcity poses a critical challenge to agricultural productivity, particularly in arid and semi-arid regions where irrigation accounts for the majority of freshwater withdrawals [1–6]. Climate change has exacerbated this issue through altered precipitation patterns and rising temperatures, emphasizing the need for sustainable crop production strategies that enhance water-use efficiency [7–10].

Sugar beet (*Beta vulgaris* L.) is recognized as one of the most important industrial crops worldwide, contributing to the production of approximately 20% of the world’s sugar [11,12]. In addition to its role in sugar production, it is widely used in various industrial sectors due to its versatility and economic potential [13–15]. However, spring cultivation of this plant is not without challenges, especially due to its high-water requirements resulting from its long growing season and high yield potential [16]. Autumn cultivation of sugar beet, on the other hand, offers several advantages over spring cultivation, making this cultivation strategy more profitable for farmers [17,18]. This planting season allows the plant to take full advantage of seasonal rainfall, thereby reducing the need for supplemental irrigation [19,20]. The lower water requirement in autumn and winter not only conserves water resources but also leads to lower production costs. Furthermore, autumn-grown sugar beet often exhibits higher economic value [21,22], making autumn cultivation an economically viable option for farmers.

Despite these advantages, autumn cultivation of sugar beet faces the significant challenge of the transition from the vegetative to the reproductive growth phase, known as bolting [17]. Bolting is a physiological process in which the plant transfers its energy from leaf and root growth to the production of a flowering stem. This shift can markedly reduce both the quality and quantity of sugar beet yield [23], as it diverts resources from the root, which is the main economic product. The severity of bolting losses can vary depending on the growth stage of the plants [17]. Bolting occurring early in the plant growth period can cause a significant reduction in RY of up to 50% [23]. Additional consequences include significant reductions in sugar yield through decreased sugar content (SC) and RY, problems with harvesting machines, blunting of the cutting blades in the sugar factory due to hardening and fibrous roots, and increased risk of weed seed dispersal [18,24]. Overall, bolting results in a crop with a high dry matter content of stem tissue and a low amount of root tissue [23], rendering them unsuitable for sugar production [25].

The phenomenon of bolting is a genetically controlled process [26,27] induced by environmental conditions such as temperature and day length in sugar beet [28]. As a result, genetic variation among genotypes leads to differential responses to cold winter temperatures [17]. Resistance to bolting is a crucial trait in this context. In bolting-resistant plants, high and prolonged cold temperatures are required for vernalization, depending on their degree of resistance. In contrast, in bolting-sensitive plants, even short-term exposure to cold temperatures can lead to vernalization [29]. Subsequently, long-day conditions in spring induce flowering and shoot production in sensitive plants [28]. Hence, accurate prediction of bolting risk is essential to optimize planting location and timing and minimize yield losses. In this regard, the vernalization-intensity model [28], developed based on cumulative hours of vernalization temperatures and variety-specific bolting thresholds and sensitivities, is a robust tool for this purpose. Furthermore, the quantitative and qualitative performance of different genotypes is significantly influenced by the interaction between plant genetic structure and the diverse climatic conditions of autumn growing environments. Consequently, it is crucial to consider the effect of GEI when selecting the most suitable genotype [30].

Accurate statistical approaches are required to dissect complex GEI patterns. The AMMI model is widely used for visualizing these interactions [31,32]; however, it relies on fixed-effect models and assumes additive environmental effects, which may lead to biased parameter estimates when these assumptions are violated [33–35]. To address these limitations, the best linear unbiased prediction (BLUP) model offers improved accuracy by treating genotype effects as random [33]. By combining the visualization power of AMMI with the predictive accuracy of BLUP, the weighted average of absolute scores (WAASB) index was introduced to quantify stability [33]. Furthermore, the WAASBY index extends this approach by simultaneously accounting for both mean performance (yield) and stability, thereby offering a comprehensive tool for genotype selection [36].

In addition to yield stability, successful breeding requires simultaneous selection for multiple agronomic traits. The MTSI [37] provides a robust framework for this purpose using mixed-model data. Based on factor analysis and the distance from an ideal genotype, MTSI allows breeders to select genotypes that exhibit high performance across various traits, such as increasing yield while decreasing bolting sensitivity, while accounting for trait correlations [38–41]. This index is particularly effective for identifying resilient genotypes in multi-environment trials (METs).

Given the urgent need to optimize water consumption in arid regions, expanding autumn sugar beet cultivation has become a strategic priority. This approach is particularly important given the increasing severity of water scarcity. Additionally, the phenomenon of bolting poses a significant limiting factor in areas with higher latitudes. Addressing these challenges is essential for the successful implementation of autumn cultivation strategies and for achieving the broader goals of enhancing sugar beet production and sustainability. In this regard, this study aimed to compare the vernalization intensity and bolting sensitivity, as well as to evaluate the quantitative and qualitative performance of different promising sugar beet genotypes under autumn cultivation conditions. The present study seeks to identify and select genotypes that can optimize yield while minimizing the adverse effects of bolting, thereby contributing to improved sustainability and productivity in water-limited environments.

## Materials and methods

### Plant materials

The plant materials used in this study consisted of 10 experimental hybrids along with two bolting-resistant control varieties, Rajah and Ratna. These varieties were obtained from the seed bank of the Sugar Beet Seed Institute (SBSI) in Karaj, Alborz, Iran. The primary breeding objective for developing these hybrids was to enhance resistance to bolting. The characteristics of the studied hybrids are presented in Table 1.

**Table 1:** Characteristics of sugar beet genotypes used in the experiment.

Row	Genotype	Row	Genotype	Row	Genotype
1	BOL239	5	BOL434	9	BOL068
2	BOL374	6	BOL435	10	BOL070
3	BOL375	7	BOL436	11	Rajah
4	BOL376	8	BOL437	12	Ratna

### Research sites

The plant materials were evaluated in three provinces Golestan, Khuzestan, and Kohgiluyeh and Boyer-Ahmad. The evaluations were conducted at the Gonbad Agricultural Research Station in Golestan, the Dezful Agricultural Research Station in Khuzestan, and the Gachsaran Agricultural Research Station in Kohgiluyeh and Boyer-Ahmad. The Gonbad station is located at 54°4’ E, 38°55’ N, at an elevation of 1390 m. The Dezful station is located at 48°26’E and 32°14’N, at an elevation of 81 m. The Gachsaran station is located at 50°50’E and 30°17’N, at an elevation of 710 m. The meteorological characteristics of the research stations during the experiment are detailed in Table 2. Complete daily temperature records are presented in S1 Table.

**Table 2:** Minimum, maximum, and mean temperatures, and total monthly rainfall in the experimental regions.

Month	Mean minimum temperature (ºC)			Mean maximum temperature (ºC)			Mean temperature (ºC)			Rainfall (mm)		
	Gonbad	Dezful	Gachsaran	Gonbad	Dezful	Gachsaran	Gonbad	Dezful	Gachsaran	Gonbad	Dezful	Gachsaran
***2022–2023***												
October	14.90	19.76	16.71	27.60	38.53	37.31	21.30	29.14	27.00	12.30	0.00	0.00
November	12.81	16.50	14.20	27.40	29.50	27.90	20.10	23.00	21.06	42.50	23.70	58.10
December	7.10	11.72	9.11	19.50	24.83	22.85	13.30	18.34	15.91	27.20	13.51	25.30
January	5.80	8.03	7.12	19.30	17.01	16.67	12.50	12.52	11.87	8.00	129.60	263.50
February	4.70	6.85	6.21	16.40	17.93	16.66	10.61	12.39	11.41	19.70	38.80	95.20
March	3.62	12.11	9.80	14.50	26.00	24.20	9.00	19.06	17.00	9.30	53.82	20.60
April	8.90	13.20	9.34	21.90	27.78	25.35	15.47	20.49	17.33	26.60	61.51	44.20
May	13.74	17.87	14.44	27.10	37.58	34.50	20.40	27.73	24.45	33.50	0.54	3.40
June	19.34	24.70	20.32	35.10	43.90	40.60	27.23	34.30	30.45	38.60	0.10	0.00
July	22.80	24.89	21.30	36.20	47.94	42.20	29.50	36.42	31.05	0.40	0.00	0.00
August	23.90	27.83	24.32	34.30	47.60	42.86	29.10	37.72	33.08	13.70	0.00	0.00
***2023–2024***												
October	18.00	21.90	17.71	31.50	38.80	37.34	24.70	30.36	27.52	50.20	0.40	0.00
November	9.90	16.82	13.47	22.51	29.91	28.91	16.21	23.37	21.20	32.60	88.80	113.50
December	6.50	10.74	7.30	16.60	23.23	23.31	11.60	16.99	15.34	42.40	47.20	20.70
January	0.90	8.99	5.70	13.90	21.74	20.62	7.40	15.36	13.20	36.60	15.30	11.10
February	1.43	7.82	6.01	13.94	21.56	19.70	7.60	14.69	12.81	89.80	22.80	122.20
March	7.30	9.83	7.12	22.51	23.22	20.74	14.96	16.52	13.82	33.30	62.60	47.90
April	9.51	14.30	10.60	24.30	29.50	25.40	16.94	21.90	18.00	55.30	83.60	174.60
May	14.40	18.85	15.70	28.90	80.34	31.20	21.60	26.83	23.45	93.80	137.70	90.50
June	21.40	23.72	19.51	35.53	45.57	41.30	28.41	34.65	30.44	0.70	0.00	0.00
July	23.07	25.90	19.89	35.81	48.20	42.68	29.43	37.00	31.00	8.70	0.00	0.00
August	24.30	27.01	21.95	38.40	49.40	43.08	31.40	37.06	33.89	24.10	0.00	0.00

Before the experiment, soil samples were collected from a depth of 0–30 cm in the fields. After air drying and passing through a 2 mm sieve, the samples were randomly selected and transferred to the soil laboratory to determine some of the physical and chemical properties of the soil, in order to determine the amount of fertilizer required. The results of the soil test are presented in Table 3. Based on these results, the amounts of the main fertilizers required were determined.

**Table 3:** Physical and chemical properties of soil in the experimental regions.

Location	Electrical conductivity (ds.m^-1^)	pH	P	K	Total N	Clay	Silt	Sand	Soil texture
			(ppm)		(%)	(%)			
***2022–2023***									
Gonbad	2.30	7.77	18.20	729	0.12	28	58	14	Silt- Clay- Loam
Dezful	1.70	7.68	6.10	104	0.07	28	40	32	Silt- Clay- Loam
Gachsaran	1.58	7.64	7.01	335	0.09	28	41	30	Silt- Clay- Loam
***2023–2024***									
Gonbad	1.65	7.74	9.20	390	0.09	28	58	14	Silt- Clay- Loam
Dezful	1.50	7.78	9.70	140	0.07	28	40	32	Silt- Clay- Loam
Gachsaran	1.60	7.54	7.00	322	0.08	28	41	30	Silt- Clay- Loam

### Experimental design and procedures

The experimental materials were evaluated in a randomized complete block design with four replications over two cropping years (2022–2023 and 2023–2024). Land preparation operations at each research station included plowing, discing, and creating furrows. Each experimental plot consisted of three rows of crops, each 10 m long. After preparing the planting beds, experimental planting was carried out at the Gonbad, Dezful, and Gachsaran research stations. In the 2022–2023 cropping year, planting occurred on October 12, September 23, and October 14, respectively, and in the 2023–2024 cropping year on October 12, September 24, and October 11, respectively. Excess seeds were initially sown, which were thinned after emergence at the 2–4 leaf stage, to achieve a final density of 120,000 plants per hectare. Irrigation was performed using furrow method. The amount of water consumed was calculated based on the evaporation rate from the class A evaporation pan, assuming an efficiency of 90%, and distributed equally across all plots. Weed, pest, and disease management practices were performed according to local recommendations and expert guidance. However, all international, national and institutional guidelines have been taken into account in various stages of experiments.

### Measurement of bolting severity and quantitative and qualitative performance

Before the onset of frost, the number of established plants in each plot was counted. After the frost period, the number of plants was counted again and recorded. Bolting was assessed by counting bolted plants by the end of June, and the bolting percentage was calculated based on the total number of plants. Since no bolting occurred at the Dezful Agricultural Research Station, this evaluation was limited to the Gonbad and Gachsaran research stations.

For quantitative and qualitative evaluations, roots from the experimental plots were collected, counted, and weighed at the time of harvesting. After washing the roots, root brie samples were prepared and examined in the quality control laboratory of the Sugar Beet Seed Institute for qualitative characteristics, including SC, *alpha-amino* nitrogen (N), and sodium (Na^+^) and potassium (K^+^) concentration [42]. Finally, the values obtained for these characteristics were used to estimate other characteristics such as molasses sugar (MS), white sugar content (WSC), and WSY based on Equations 1–3, respectively [43]:


$$MS=\text{0}.\text{0343}\left(K^{+}+{Na}^{+}\right)+\text{0}.\text{094}(alpha\;amino\;N)-\text{0}.\text{31}$$ (1)

WSC=SC−(MS+0.6) (2)

WSY=WSC×RY (3)

where MS is molasses sugar (%), K^+^ is potassium (meq.100 g^-1^), Na^+^ is sodium (meq.100 g^-1^), *alpha-amino-*N is nitrogen (meq.100 g^-1^), WSC is white sugar content (%), SC is sugar content (%), WSY is white sugar yield (t. ha^-1^) and RY is root yield (t. ha^-1^).

### Statistical analysis

The vernalization intensity experienced by genotypes was calculated using the minimum and maximum daily temperatures obtained from the nearest meteorological stations to the research stations. For this purpose, the diurnal and hourly temperatures were estimated using Equation 4 [28]:


$$T=f(h)=T_{m}+T_{a}\times sin(\pi /\text{1}\text{2}\times (h-h_{x}+\text{6}))$$ (4)

In this equation, T is the hourly temperature, h is the hour of the day, $T_{m}$ is the mean daily temperature, $T_{a}$ is the daily temperature range, and $h_{x}$ is the hour at which the maximum daily temperature occurs. Vernalization weighting for each hourly temperature was performed based on the cumulative number of hours between planting time and the end of June with temperatures between 0 and 13°C [28,44,45], using Equation 5 [28]:


$$y\;=\;-\text{1}.\text{2}\text{5}\text{6}+(\text{1}.\text{2}\text{6}\text{0}+\text{0}.\text{1}\text{3}\text{1}x)\times {\text{0}.\text{9}\text{3}\text{5}\text{7}}^{x}$$ (5)

In this equation, y is the vernalization weight and x is the hourly temperature. Days with maximum temperatures exceeding 23°C were excluded to account for vernalization reversal [28,46,47] were removed from the model. Vernalization intensity was then obtained from the sum of the weighted cumulative vernalization hours between planting and the end of June. Finally, the VT and bolting sensitivity parameters were estimated using biphasic linear regression fitted to the bolting pattern of individual genotypes [28].

Normality of quantitative and qualitative traits was tested using the Shapiro–Wilk test, and the assumption of homogeneity of experimental error variance across environments (two years and three locations) was checked using the Levene’s test. After confirming the homogeneity of experimental error variance, a combined analysis of variance was performed for the traits WSY, RY, SC, and concentrations of Na^+^, K^+^, and N using Equation 6 [48]:


$$Y_{ijk}=\mu +E_{i}+{B_{k}(E}_{j})+G_{j}+{GEI}_{ij}+e_{\text{ij}k}$$ (6)

where μ is the overall mean, $E_{i}$ is the effect of the $i^{th}$ environment, $G_{j}$ is the effect of the $j^{th}$ genotype, ${GEI}_{ij}$ is the interaction effect of the $i^{th}$ level of environment with the $j^{th}$ level of genotype, $B_{k}$ is the effect of the $k^{th}$ block within the $i^{th}$ environment, and $e_{ijk}$ is experimental error.

Comparison of the traits of WSY, RY, concentrations of Na^+^, K^+^, and N among six environments was performed using Fisher’s least significant difference (LSD) test at a 5% probability level. Given that the GEI was effective on the two traits of WSY and RY, additional studies of GEI were conducted only for these traits. For this purpose, to combine the AMMI and BLUP features, the scores of the aforementioned models for WSY and RY of each experimental genotype were first calculated. Then, using the WAASB based on Equation 7, the scores of the AMMI and BLUP models were combined [33]:


$${\text{WAAS}B}_{\text{i}}=\frac{\sum_{\text{k}=\text{1}}^{\text{P}}\;\left|{\text{IPCA}}_{\text{ik}}\times {\text{EP}}_{\text{k}}\right|}{\sum_{\text{k}=\text{1}}^{\text{P}}\;{\text{EP}}_{\text{k}}}$$ (7)

where (Equation 7) ${\text{WAASB}}_{\text{i}}$ is the weighted average of absolute scores of the $i^{th}$ genotype or environment; ${\text{IPCA}}_{\text{ik}}$ is the absolute score of the $i^{th}$ genotype or environment in the $k^{th}$ interaction principal component (IPC); and ${\text{EP}}_{\text{k}}$ is the magnitude of the variance explained by the $k^{th}$ IPC. Given that the attainment of high-performing, stable genotypes is a primary objective, the individual contributions of GEI were examined using a BLUP matrix. Following this analysis, the WAASBY index, which measures both yield average and stability, was calculated for each genotype as per Equation 8 [33]:


$${\text{WAAS}BY}_{\text{i}}=\frac{\left({rG}_{g}\times \theta_{Y}\right)+({rW}_{g}\times \theta_{S})}{\theta_{Y}+\theta_{S}}$$ (8)

where ${\text{WAAS}BY}_{\text{i}}$ is the superiority index with different weights between yield and stability for the $g^{th}$ genotype; $\theta_{Y}$ and $\theta_{S}$ are the weights for yield and stability, respectively; ${rG}_{g}$ and ${rW}_{g}$ are the rescaled values of the $g^{th}$ genotype for yield and WAASB, respectively.

To identify the most superior sugar beet genotypes, a comprehensive evaluation of key traits such as RY, SC, N, Na^+^, and K^+^ is necessary. To assess the stability of these traits simultaneously, the MTSI was calculated using Equation 9 [37].


$${MTSI}_{i}={\left[\sum_{j=\text{1}}^{f}\;{\left(\gamma_{ij}-\gamma_{j}\right)}^{\text{2}}\right]}^{\text{0}.\text{5}}$$ (9)

where ${MTSI}_{i}$ is the multi-trait stability index of the genotype *i*, $\gamma_{ij}$ is the score of the genotype *i* in the factor *j*, and $\gamma_{j}$ is the score of the ideal genotype in the factor *j*. The scores were calculated using factor analysis for genotypes and traits. Finally, stable genotypes were selected based on positive selection differentials for traits intended to increase and negative selection differentials for traits intended to decrease.

## Results

**Fig 1:**
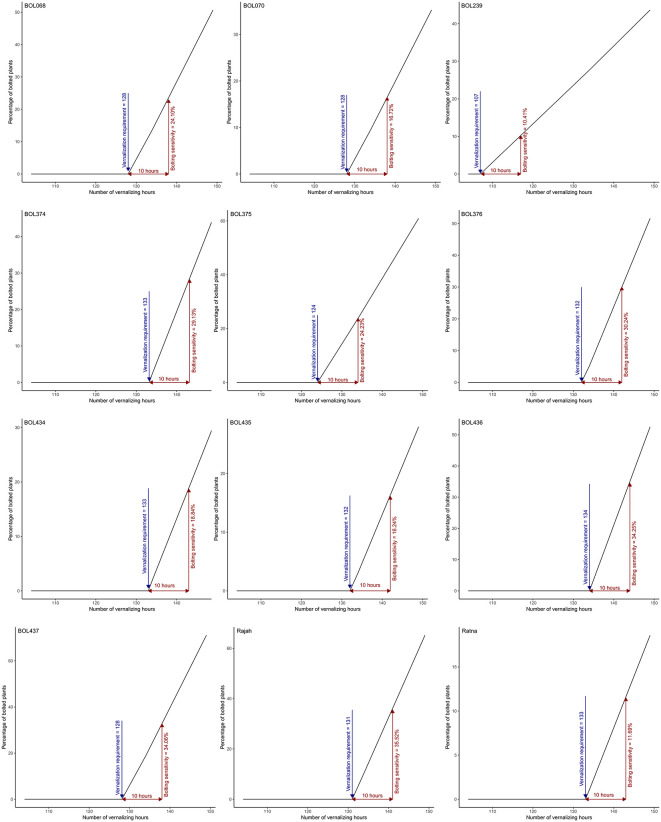
Vernalization-intensity bolting model parameters in sugar beet genotypes. Number of vernalizing hours from sowing. Increase in the proportion of bolted plants for each 10 vernalizing-hour increment above the threshold requirement.

The results of the VT analysis for the experimental genotypes (Fig 1) showed clear differences among genotypes. Genotype BOL436 required the highest VT, with 134 vernalizing hours needed to initiate bolting. This was followed by genotypes BOL374 and BOL434, as well as the control variety Ratna, each requiring 133 vernalizing hours. Genotypes BOL376 and BOL435 ranked next with VT values of 132 hours. The control variety Rajah required 131 hours of vernalization (Fig 1). Genotypes BOL437, BOL068, and BOL070 required 128 vernalizing hours. The BOL375 genotype ranked sixth with 124 hours of vernalization. The lowest VT value was observed in genotype BOL239, which required 107 vernalizing hours (Fig 1).

Bolting sensitivity was estimated based on the percentage increase in bolting associated with each 10-hour increase in VT above the genotype-specific threshold (Fig 1). The experimental genotypes showed different levels of bolting sensitivity. The BOL239 genotype and the control variety Ratna showed the lowest bolting sensitivity, with 10.41% and 11.69% bolting per 10-hour increase in VT, respectively. Genotypes BOL435, BOL070, BOL434, BOL068, and BOL375 showed moderate sensitivity, with bolting increases of 16.24%, 16.72%, 18.84%, 24.10%, and 24.23%, respectively, per 10-hour VT increment. The highest bolting sensitivity was observed in genotypes BOL374, BOL376, BOL437, and BOL436, along with the control variety Rajah, with increases of 29.13%, 30.24%, 34.06%, 34.25%, and 35.52%, respectively (Fig 1).

The combined analysis of variance for WSY, RY, SC, and the concentrations of Na^+^, K^+^, and N is presented in Table 4. Both environmental and genotypic main effects were significant at the 1% probability level for all six traits. However, GEI was significant only for WSY and RY (1% level), and was not significant for SC or for Na ⁺ , K ⁺ , and N concentrations. The likelihood ratio test (Table 4) confirmed that environmental effects were significant for all traits at the 1% probability level. Genotypic effects were significant for SC and for Na^+^ and K^+^ concentrations at the 1% probability level. The GEI had a significant effect on only two traits—WSY and RY—at the 1% probability level.

**Table 4:** Combined analysis of variance and likelihood ratio test for studied traits in sugar beet genotypes.

Source of variation	df	White sugar yield		Root yield		Sugar content		Na^+^ concentration		K^+^ concentration		N concentration	
		MS.	Pro.	MS.	Pro.	MS.	Pro.	MS.	Pro.	MS.	Pro.	MS.	Pro.
Environment	5	897.35**	–	30275.91**	–	152.10**	–	2196227.09**	–	4003709.87**	–	420693.09**	–
Replication (environment)	18	8.18	–	219.45	–	1.89	–	69556.79	–	208227.63	–	19177.00	–
Genotype	11	9.03**	–	571.08**	–	4.50**	–	137949.22**	–	306964.78**	–	7825.89**	–
Genotype-environment	55	5.86**	–	262.65**	–	0.58^ns^	–	12550.10^ns^	–	48297.01^ns^	–	3674.48^ns^	–
Residuals	198	3.10	–	142.11	–	0.68	–	15366.60	–	37178.53	–	3232.17	–
Total	342	17.52	–	639.30	–	3.05	–	53139.47	–	116424.79	–	10464.62	–
Likelihood ratio test													
Environment		3.10**	79.05	142.11**	76.78	0.65**	80.38	14754.32**	74.19	37178.54**	73.27	3232.17**	91.93
Genotype		0.14^ns^	3.37	12.85^ns^	6.94	0.16**	19.62	5133.12**	25.81	10777.86**	21.24	172.98^ns^	4.92
Genotype-environment		0.69**	17.58	30.13**	16.28	0.00^ns^	0.00	0.00^ns^	0.00	2779.58^ns^	5.48	110.58^ns^	3.14
Genetic variance component													
$\delta_{\text{P}}^{\text{2}}$		3.92		185.09		0.82		19887.44		50735.99		3515.73	
${\text{h}}_{\text{mg}}^{\text{2}}$		0.35		0.54		0.85		0.89		0.84		0.53	
${\text{R}}^{\text{2}}$ Genotype-environment		0.18		0.16		0.00		0.00		0.05		0.03	
Accuracy		0.59		0.73		0.92		0.94		0.92		0.73	

MS: mean of square, Pro: Proportion (%), **: 1% probability level of significance, *: 5% probability level of significance, ns: non-significant.

The components of genetic variance were estimated for each of the studied traits (Table 4). Environmental variance accounted for the highest proportion in all six traits, accounting for 79.05%, 76.78%, 80.38%, 74.19%, 73.27%, and 91.93% of the phenotypic variance in WSY, RY, SC, and concentrations of Na^+^, K^+^, and N, respectively (Table 4). For WSY and RY, genotypic variance accounted for the lowest share of phenotypic variance, while the opposite was true for SC and concentrations of Na^+^, K^+^, and N. The genotypic variance for the six traits was 3.37%, 6.94%, 19.62%, 25.81%, 21.24%, and 4.92%, respectively (Table 4). GEI variance accounted for the second-largest proportion of phenotypic variation in WSY and RY, explaining 17.58% and 16.28%, respectively. It ranked third for SC and concentrations of Na^+^, K^+^, and N, explaining 0%, 0%, 5.48%, and 3.14% of the phenotypic variation, respectively (Table 4).

Heritability estimates indicated moderate heritability for WSY (0.35), whereas Na⁺ concentration, SC, K⁺ concentration, RY, and N concentration exhibited higher heritability values of 0.89, 0.85, 0.84, 0.54, and 0.53, respectively (Table 4). Genotypic correlations between environments were low for all traits (Table 4). Selection accuracy was high for Na⁺ concentration, SC, K⁺ concentration, RY, N concentration, and WSY, with corresponding coefficients of 0.94, 0.92, 0.92, 0.73, 0.73, and 0.59 (Table 4).

**Fig 2:**
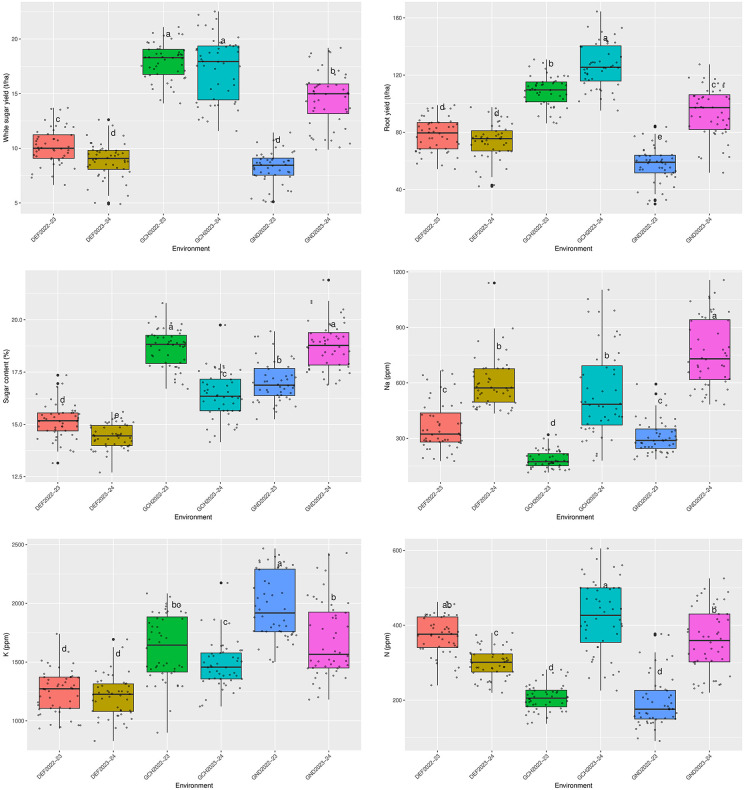
Comparison of white sugar yield, root yield, sugar content, and concentrations of Na^+^, K^+^ and *alpha amino* N among six environments. Different lowercase letters indicate statistically significant differences as determined by Fisher’s least significant difference (LSD) test at the 5% probability level. DEF2022-23: Dezful in 2022-2023, DEF2023-24: Dezful in 2023-2024, GCH2022-23: Gachsaran in 2022-2023, GCH2023-24: Gachsaran in 2023-2024, GND2022-23: Gonbad in 2022-2023 and GND2023-24: Gonbad in 2023-2024.

**Fig 3:**
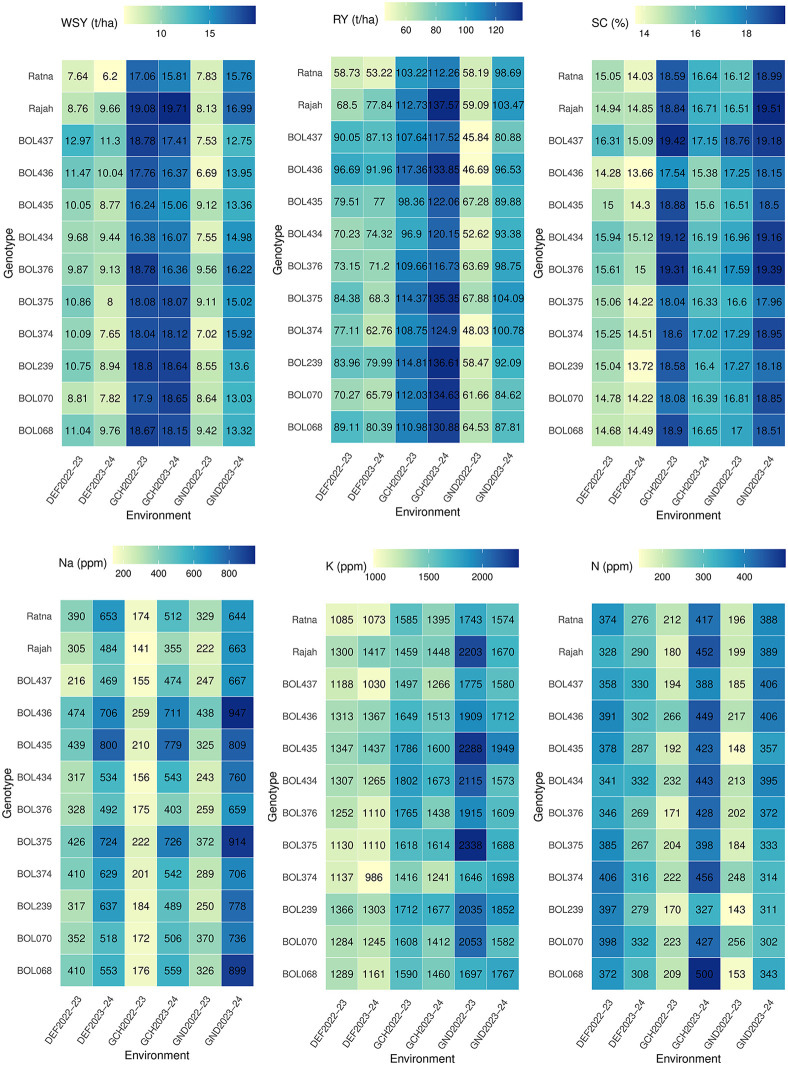
Performance of experimental sugar beet genotypes: white sugar yield, root yield, sugar content, and concentrations of Na^+^, K^+^ and *alpha amino* N among six environments. DEF2022–23: Dezful in 2022–2023, DEF2023–24: Dezful in 2023–2024, GCH2022–23: Gachsaran in 2022–2023, GCH2023–24: Gachsaran in 2023–2024, GND2022–23: Gonbad in 2022–2023 and GND2023–24: Gonbad in 2023–2024.

The mean values of six traits—WSY, RY, SC, and concentrations of Na^+^, K^+^, and N—in six environments (three locations over two years) are presented in Fig 3. The values for each trait varied among the tested environments. In terms of WSY, GCH2022−23 and GCH2023−24 had the highest yields, with averages of 17.96 and 17.37 t. ha^-1^, respectively. These yields were significantly higher than those in GND2023−24 and DEF2022−23, which had average yields of 14.57 and 10.16 t. ha^-1^, respectively. A significant difference was also observed between these two environments. In contrast, GND2022−23 and DEF2023−24 had the lowest yields, with averages of 8.26 and 8.89 t. ha^-1^, respectively. Regarding RY, GND2023−24 ranked first with an average of 126.87 t. ha^-1^, followed by GCH2022−23, GND2022−23, DEF2022−23, and DEF2023−24 with averages of 108.90, 94.24, 78.47, and 74.16 t. ha^-1^, respectively. GND2022−23 produced the lowest RY with a yield average of 57.83 t. ha^-1^. The highest level of SC was observed in GND2023−24 and GCH2022−23, with averages of 18.78% and 18.66%, respectively. This was followed by GND2022−23, GCH2023−24, and DEF2022−23 with averages of 17.06%, 16.40%, and 15.16%, respectively, which were significantly different from each other. DEF2023−24 had the lowest level of SC with 14.43% (Fig 2).

Impurities in the root, especially soluble mineral salts (Na^+^ and K^+^) and N, are undesirable traits that make sugar extraction difficult. Roots produced in GND2023−24 had the highest Na^+^ concentration, with an average of 765.11 ppm (Fig 2). Following that, DEF2023−24, GCH2023−24, DEF2022−23, and GND2022−23 were ranked, with their Na^+^ concentrations varying from 305.77 to 599.85 ppm. There was a significant difference between the first two environments and the second two environments. Roots produced in GCH2022−23 showed the lowest Na^+^ concentration, with an average of 185.50 ppm (Fig 2). Comparison of K^+^ concentration indicated that the roots produced in GND2022−23 had the highest concentration, with an average of 1976.48 ppm. GND2023−24, GCH2022−23, and GCH2023−24 were ranked next, with averages of 1687.73, 1623.87, and 1477.98 ppm, respectively. DEF2023−24 and DEF2022−23 had lower K^+^ concentrations, with averages of 1208.84 and 1249.81 ppm, respectively (Fig 2). Regarding the N concentration, the roots of GCH2023−24 and DEF2022−23 had the highest amounts, with averages of 425.58 and 372.87 ppm, respectively. Following that, GND2023−24 and DEF2023−24 had averages of 359.53 and 298.97 ppm, respectively, which were also significantly different from each other. The lowest concentration was observed in GND2022−23 and GCH2022−23, with averages of 195.32 and 206.15 ppm, respectively (Fig 2).

The functional value of different sugar beet genotypes for each of the six traits—WSY, RY, SC, and concentrations of Na^+^, K^+^, and N—in each of the six experimental environments is shown in Fig 3. The results show that different environments have varying effects on the studied traits of the experimental genotypes, and genotypic responses differ based on genetic structure. Comparison of WSY among genotypes in each environment showed that in DEF2022−23 and DEF2023−24, the genotypes BOL473 and BOL436 had significantly higher yields than the control varieties Rajah and Ratna, as well as the other experimental genotypes. In DEF2022−23, BOL473 and BOL436 had average yields of 12.97 and 11.47 t. ha^-1^, respectively. In DEF2023−24, their yields were 11.30 and 10.03 t. ha^-1^, respectively. On the contrary, in GCH2022−23 and GCH2023−24, all genotypes and control varieties except the genotype BOL435 were in the same group without significant differences, with yields ranging between 15.81 and 19.70 t. ha^-1^. In GND2022−23 and GND2023−24, a different trend was observed. In GND2022−23, genotypes BOL376, BOL068, BOL435, and BOL375 had higher yields than the control varieties Rajah and Ratna and other experimental genotypes, with average yields of 9.56, 9.42, 9.12, and 9.11 t. ha^-1^, respectively. In GND2023−24, all experimental genotypes and control varieties except the BOL437 genotype recorded the highest yields, ranging from 13.03 to 16.99 t. ha^-1^, without significant differences (Fig 3).

The observed trend for RY (Fig 3) was largely similar to that of WSY. In DEF2022−23 and DEF2023−24, RY varied significantly among genotypes, ranging from 58.73 to 96.69 t. ha^-1^ in DEF2022−23 and from 53.22 to 91.96 t. ha^-1^ in DEF2023−24. The highest RY was recorded for genotypes BOL436 and BOL437, with average yields of 96.69 and 90.05 t. ha^-1^ in DEF2022−23, and 91.96 and 87.13 t. ha^-1^ in DEF2023−24, respectively. In GCH2022−23, all genotypes except genotypes BOL434 and BOL435 had high yields, as did all genotypes except the control variety Ratna in GCH2023−24. Yields ranged from 103.22 to 117.36 t. ha^-1^ in GCH2022−23 and from 116.73 to 137.57 t. ha^-1^ in GCH2023−24. In GND2023−24, although no statistically significant difference was observed between genotypes, the genotypes showed different yields in GND2022−23, with the highest RYs belonging to genotypes BOL375 and BOL435, with averages of 67.87 and 67.28 t. ha^-1^, respectively.

The results of SC (Fig 3) showed that the genotypes BOL437 and BOL434 had the highest SC, with values of 16.31% and 15.94% in DEF2022−23, and 15.09% and 15.12% in DEF2023−24, respectively. These genotypes had a significant advantage over the control varieties Ratna and Rajah, which had 15.05% and 14.94% in DEF2022−23, and 14.02% and 14.85% in DEF2023−24, respectively. In GCH2022−23, the genotypes BOL437 and BOL376 had the highest SC, with averages of 19.42% and 19.31%, respectively. In GCH2023−24, the BOL437 genotype again showed the highest SC, with an average of 17.15%, which was significantly higher than the control varieties. In GND2023−24, no significant difference was observed; however, in GND2022−23, BOL437 recorded the highest content (18.76%), significantly outperforming control varieties Rajah (16.51%) and Ratna (16.12%).

In DEF2022−23 and DEF2023−24, genotype BOL437 had the lowest Na^+^ concentration, with averages of 216 and 469 ppm, respectively. In GCH2022−23, in addition to the genotype BOL437, which had an average of 188 ppm, genotype BOL434 and the control variety Rajah also had the lowest Na^+^ concentrations, with averages of 156 ppm and 141 ppm, respectively. In GCH2023−24, genotype BOL376 and the control variety Rajah had the lowest Na^+^ concentrations, with averages of 403 and 355 ppm, respectively. In GND2022−23, genotypes BOL434, BOL437, BOL239, BOL376, and the control variety Rajah showed the lowest Na^+^ concentrations, with averages of 243, 247, 250, 259, and 222 ppm, respectively. In GND2023−24, only the control variety Rajah had the lowest Na^+^ concentration, with an average of 644 ppm (Fig 3).

In DEF2022−23, no significant difference was observed between genotypes in terms of K^+^ concentration; all genotypes and the control varieties Rajah and Ratna were placed in the same statistical group, with an average K^+^ concentration ranging from 1085 to 1366 ppm. However, in DEF2023−24, genotype BOL374 had a significantly lower concentration, with an average of 986 ppm, compared to the control varieties Rajah and Ratna and other experimental genotypes. This genotype (BOL374) also had the lowest K^+^ concentration in GCH2022−23 and GCH2023−24, with averages of 1416 and 1241 ppm, respectively. Genotype BOL374 maintained its significant superiority over the control varieties and experimental genotypes in GND2022−23, recording the lowest K^+^ concentration with an average of 1646 ppm. However, in GND2023−24, this superiority was lost, and all experimental genotypes and control varieties were placed in the same statistical group, with K^+^ concentrations ranging from 1573 to 1949 ppm (Fig 3).

Based on the results obtained from DEF2022−23, no significant difference was observed between genotypes in terms of N concentration. However, in DEF2023−24, genotypes BOL375 and BOL376 had low N concentrations, with averages of 267 and 269 ppm, respectively. In GCH2022−23, two genotypes, BOL239 and BOL376, had averages of 170 and 171 ppm, respectively. In GCH2023−24, genotype BOL239 had an average of 327 ppm, which was significantly lower than the control varieties. In GND2023−24, although no statistically significant difference was observed between genotypes, the genotypes showed different N concentrations in GND2022−23. The lowest concentrations corresponded to genotypes BOL239, BOL435, and BOL068, with averages of 143, 148, and 153 ppm, respectively (Fig 3).

Given the significant effect of GEI on WSY and RY, a multiplicative interaction analysis was performed, with results presented in Table 5. The number of significant IPCs varied between the two traits. For WSY, the first two IPCs were significant at the 1% and 5% probability levels, respectively, cumulatively explaining 75.20% of the GEI variation. In contrast, for RY, the first IPC alone was significant at the 1% probability level, and accounted for 57.60% of the interaction variation. Although the first one or two IPCs captured the majority of the variation, relying solely on them might overlook specific interaction patterns associated with certain genotypes. Consequently, the WAASB index was employed as a robust quantitative measure of stability, as it integrates the variance of all significant interaction components.

**Table 5:** Analysis of variance for genotype- environment interaction in white sugar yield and root yield based on the AMMI model in sugar beet genotypes.

Source of variation	df	White sugar yield			Root yield		
		Mean of square	Relative variance (%)	Cumulative variance (%)	Mean of square	Relative variance (%)	Cumulative variance (%)
PC1	15	11.30**	52.60	52.60	554.49**	57.60	57.60
PC2	13	5.61*	22.60	75.20	184.71^ns^	16.60	74.20
PC3	11	4.76^ns^	16.20	91.40	187.62^ns^	14.30	88.50
PC4	9	2.15^ns^	6.00	97.40	127.29^ns^	7.90	96.40
PC5	7	1.17^ns^	2.60	100.00	73.94^ns^	3.60	100.00

PC: principal component, **: 1% probability level of significance, *: 5% probability level of significance, ns: non-significant.

**Fig 4:**
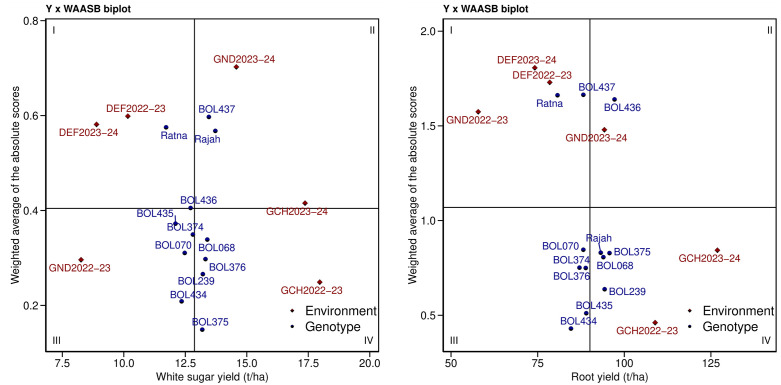
Biplot of sugar beet genotypes, white sugar yield and root yield with weighted average absolute scores of the best linear unbiased predictions (WAASB). DEF2022-23: Dezful in 2022-2023, DEF2023-24: Dezful in 2023-2024, GCH2022-23: Gachsaran in 2022-2023, GCH2023-24: Gachsaran in 2023-2024, GND2022-23: Gonbad in 2022-2023 and GND2023-24: Gonbad in 2023-2024.

Fig 4 shows a biplot with yield traits (WSY and RY) on the horizontal axis and WAASB on the vertical axis. In this biplot, the vertical line in the middle represents the total yield average across the experimental environments. Genotypes and environments to the right of this line show yield values higher than the total average, while genotypes and environments to the left have yield values lower than the total average. The horizontal axis in the middle of the biplot represents the WAASB average. From the intersection of this axis with the vertical axis, the biplot is divided into four parts. Genotypes in different quadrants of the biplot can be classified based on their stability across different environments. In the first quadrant of the biplot for WSY, the genotype BOL436 and control variety Ratna are found along with DEF2022−23 and DEF2023−24. Similarly, in the first quadrant of the biplot for RY, genotype BOL437 and the control variety Ratna are found along with DEF2022−23, DEF2023−24, and GND2022−23. These genotypes and environments had high WAASB and lower yields than the total average.

In the second quadrant of the WSY biplot, genotype BOL437 and control variety Rajah are located along with GND2023−24 and GCH2023−24. The same quadrant in the RY biplot includes the genotype BOL436 and GND2023−24. These genotypes and environments have high WAASB and yields higher than the total average. In the third quadrant of the WSY biplot, genotypes BOL434, BOL070, BOL374, and BOL435 are located along with GND2022−23. Similarly, in the RY biplot, genotypes BOL434, BOL435, BOL376, BOL374, and BOL070 are found in this quadrant. These genotypes and environments have lower WAASB and yields. In the fourth quadrant of the WSY biplot, genotypes BOL375, BOL239, BOL376, and BOL068 are present along with GCH2022−23. In the fourth quadrant of the RY biplot, genotypes BOL239, BOL068, and BOL375 are present along with control variety Rajah and GCH2022−23 and GCH2023−24. These genotypes and environments have low WAASB and yields higher than the total average (Fig 4).

**Fig 5:**
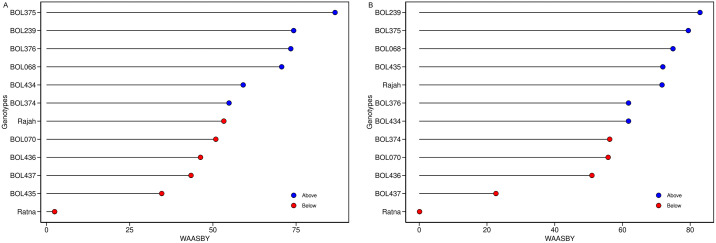
Estimated WAASBY with equal weights for white sugar yield (A) /root yield (B) and WAASB in sugar beet genotypes.

Fig 5 illustrates the ranking and selection of genotypes based on a 50:50 weighting for the WAASB stability index and yield (WSY or RY). Blue circles indicate WAASBY scores above the overall average, while red circles indicate scores below the average. In terms of WSY (Fig 5A), six genotypes—BOL375, BOL239, BOL376, BOL068, BOL434, and BOL374—had WAASBY scores higher than the average. Genotype BOL375 ranked first, followed by BOL239, BOL376, and BOL068, which showed relatively higher scores compared to the others. For RY (Fig 5B), the results were largely similar, with only the ranking order differing. Six genotypes—BOL239, BOL375, BOL068, BOL435, BOL376, and BOL434—along with the control variety Rajah, exhibited WAASBY scores above the average. Among them, genotype BOL239 ranked first, followed by BOL375, BOL068, and BOL435. Based on these results, genotypes BOL375, BOL239, BOL376, and BOL068 were identified as suitable for both WSY and yield stability. Similarly, genotypes BOL239, BOL375, BOL068, and BOL435 were found suitable for RY and high stability.

Table 6 presents the results of the factor analysis. Factors with eigenvalues greater than 1 were retained, and their variance contribution was expressed as a percentage to indicate their relative importance. Three independent factors were identified, explaining a total of 84.84% of the variation in the data. The first factor (eigenvalue 2.54) accounted for 43.42% of the total variance and was characterized by large negative factor loadings for SC and Na^+^ concentration. The second factor (eigenvalue 1.44) explained 23.23% of the variation, featuring a large positive loading for N concentration and a large negative loading for bolting percentage. The third factor (eigenvalue 1.10) accounted for 18.18% of the variation, with a large negative loading for RY and a large positive loading for K^+^ concentration.

**Table 6:** Prediction of selection differential for studied traits based on MTSI index.

Variable	FA1	FA2	FA3	Communality	Uniqueness’s	Goal	h^2^	SD (%)	SG (%)
Root yield	0.09	−0.15	−0.95	0.94	0.06	increase	0.54	−0.90	−0.49
Sugar content	−0.91	0.06	0.18	0.86	0.13	increase	0.85	1.17	0.99
${\text{Na}}^{+}$	−0.92	−0.07	0.16	0.88	0.12	decrease	0.89	−9.54	−8.52
${\text{K}}^{+}$	−0.26	−0.29	0.88	0.92	0.08	decrease	0.84	3.95	3.33
*alpha amino* N	0.26	0.83	−0.07	0.76	0.24	decrease	0.53	−3.67	−1.95
Bolting	0.43	−0.74	−0.01	0.73	0.27	decrease	0.39	−2.85	−1.10
Mean	–	–		0.85	0.15	–	–	–	–
Eigenvalues	2.54	1.44	1.1	–	–	–	–	–	–
Variance (%)	42.43	23.92	18.49	–	–	–	–	–	–
Cum. variance (%)	42.43	66.35	84.84	–	–	–	–	–	–

FA: Factor, h^2^: Heritability, SD: Selection differential, SG: Selection gain, Cum. variance: cumulative variance.

**Fig 6:**
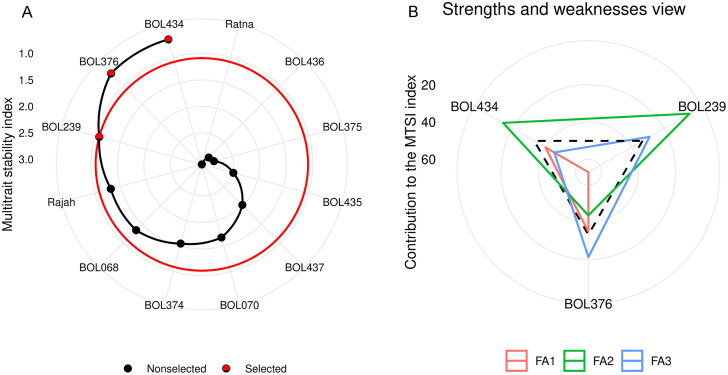
Ranking of sugar beet genotypes in ascending order based on the multi-trait stability index (A) and strengths and weaknesses of selected sugar beet genotypes as the ratio of each factor in the calculated multi-trait stability index (B). FA: Factor.

The MTSI for the studied genotypes was calculated based on the scores of these three factors. Fig 6A ranks the experimental genotypes from highest to lowest MTSI; genotypes with higher index values are positioned in the center, while those with lower values are closer to the outer edge. With a selection pressure of 25%, genotype BOL434 ranked first, followed by BOL376 and BOL239. These were identified as the most stable genotypes across all traits, including RY, SC, bolting percentage, and concentrations of Na^+^, K^+^, and N.

Comparing the trait means of the selected genotypes (based on MTSI) with the grand mean of all experimental genotypes revealed specific trends. The mean values for SC and K^+^ concentration increased in the selected group, while RY, bolting percentage, Na^+^, and N concentrations decreased. The increase in SC aligns with breeding goals, whereas the increase in K^+^ concentration contrasts with the objective of reducing impurities. However, the reduction in RY was negligible (0.90%). Ideally, bolting percentage and concentrations of Na^+^ and N should be reduced, and the selected genotypes achieved significant gains in these traits. Overall, the selected genotypes yielded favorable selection differentials and progress for all traits except RY and K^+^ concentration (Table 6). Notably, all traits in the selected genotypes, except for bolting percentage, exhibited high heritability.

Fig 6B illustrates the strengths and weaknesses of the selected genotypes based on the contribution of each factor to the MTSI. A lower contribution by a factor (indicated by proximity to the outer edge) implies that the traits associated with that factor are closer to the stable ideotype. The dotted line represents the theoretical value if all factors contributed equally. Consequently, genotypes contributing less to a specific factor are closer to the ideal for the traits within that factor. For instance, genotypes BOL376 and BOL434 had the lowest values in the first factor, indicating they are close to the ideal for SC and Na^+^ concentration (which had the highest loadings in Factor 1). Similarly, genotypes BOL239 and BOL434 had the lowest contribution to the second factor, placing them near the ideal for bolting percentage and N concentration. Finally, genotypes BOL376 and BOL239 showed the lowest values in the third factor, indicating proximity to the ideal genotype for RY and K^+^ concentration.

## Discussion

The results of the VT study provided valuable information on the bolting behavior of the experimental genotypes and their bolting sensitivity. The BOL436 genotype showed the highest VT, followed by BOL374, BOL434, and the control variety Ratna. This aligns with the negative correlation typically observed between high vernalization requirements and early bolting [49]. Therefore, the higher the VT, the greater the resistance to bolting. However, another parameter of the vernalization-intensity model is bolting sensitivity [28], which is very decisive when selecting varieties for autumn cultivation of sugar beet. As mentioned, the BOL436 genotype had the highest VT but was also very sensitive to bolting. In contrast, the control variety Ratna and genotypes BOL435 and BOL434 showed the least sensitivity, making them preferred for cultivation. These findings contrast with some previous reports [17] that suggested a more uniform vernalization requirement, indicating a complex genetic and environmental interaction likely attributed to the diverse genetic backgrounds of the studied genotypes.

The results of the vernalization-intensity model have significant implications for farmers and breeders. Understanding the vernalization requirements can help optimize cultivation strategies and minimize performance losses [28]. A potential advantage of high vernalization requirements is the reduced risk of early bolting under fluctuating winter temperatures [17,23,50]. The vernalization-intensity model proposed by Milford, Jarvis [28] is the most suitable model to provide this valuable information.

The combined analysis of variance confirmed significant effects of genotype, environment, and GEI on key yield traits, particularly WSY and RY. The significant GEI emphasizes that environmental factors heavily influence the performance of high-yielding genotypes, consistent with previous studies [51,52]. These findings align with reports attributing significant phenotypic variations to fluctuating environmental conditions and genetic background [36,53]. Consequently, evaluating genotypes across diverse environments is crucial to account for differential genotypic responses, as highlighted by Sadeghzadeh Hemayati, Saremirad [54] and Saremirad and Taleghani [53]. Understanding these interactions is vital for optimizing breeding strategies and minimizing yield losses due to environmental variability.

While GEI is often viewed as a challenge, it offers opportunities to identify genotypes with specific adaptability. This enables the development of specialized varieties tailored to distinct climatic zones, maximizing both yield and quality. For instance, in autumn sugar beet cultivation, genotypes adapted to cooler, high-rainfall climates can be targeted for regions with analogous environmental conditions. Consequently, multivariate statistical methods are instrumental in revealing and interpreting these complex interactions [36].

The LRT results confirmed the combined analysis of variance, indicating that GEI significantly affected only WSY and RY. This highlights that the phenotypic expression of these polygenic traits is heavily influenced by the interplay between genetic background and environmental conditions [55]. Such interactions can complicate selection response [56], necessitating robust estimation methods like BLUP [37]. Consequently, genetic variance components were estimated using the REML/BLUP method. Environmental variance accounted for the largest proportion of phenotypic variance across all traits. For WSY and RY, genotypic variance contributed the least, whereas the opposite was observed for SC and impurities (Na^+^, K^+^, and N). Since the ratio of genetic to environmental variance is lower for yield traits, selection efficiency decreases, making the identification of desirable genotypes more challenging [57].

The relatively low variance of GEI indicated that this effect had a minor impact on phenotypic expression compared to environmental effects, resulting in moderate rank fluctuations across environments. Indeed, when GEI variance is low relative to the environmental variance, it implies that phenotypic plasticity is driven more by uniform environmental responses than by genotype-specific deviations [58]. This stability is crucial for trait selection efficiency [59]. The results of the study on winter sugar beet cultivation by Taleghani, Saremirad [50] confirmed that the environment had the greatest impact on WSY, followed by genotype, which had the most explained variance. The GEI accounted for the least variation in the data. In the study by Basafa and Taherian [60], the variance explained by GEI was 84.7%. In the study by Mostafavi and Saremirad [61], genotype caused the most variation in yield, indicating the high diversity of genotypes. Hassani, Mahmoudi [36] showed that environmental variance accounted for the most variation in both RY and WSY, followed by GEI variance in second place and genotypic variance in third place, which was consistent with the results obtained for the present study.

The AMMI analysis revealed that the first two principal components captured the majority of the variation in GEI for both traits. Although the first one or two PCs explained the behavior of most genotypes, significant residual variation remained for others [36,62]. Consequently, the WAASB index was employed as a robust quantitative measure of stability, as it integrates the variance of all significant interaction principal components rather than relying solely on the first axis [41].

The WAASB biplot analysis provided a detailed categorization of genotypes based on their yield-stability relationship. Genotypes BOL436 and the control variety Ratna exhibited high WAASB scores combined with below-average yields for WSY, indicating high instability and poor performance; thus, they are not recommended. In contrast, genotype BOL437 and the control variety Rajah (for WSY), as well as BOL436 (for RY), displayed high yields but also high WAASB scores. While they contributed significantly to GEI, their high mean performance suggests they could be suitable for environments with favorable growing conditions, despite their volatility. On the other hand, genotypes such as BOL434, BOL070, and BOL374 were characterized by low WAASB scores but also low yields. Although highly stable and insensitive to environmental fluctuations, their lack of productivity makes them less desirable for commercial cultivation. Crucially, genotypes BOL375, BOL239, BOL376, and BOL068 were identified as the most desirable candidates. They exhibited the ideal combination of low WAASB scores (high stability) and above-average yields for both WSY and RY. Consequently, these genotypes are recommended for broad cultivation across diverse environments. The successful identification of these superior genotypes highlights the analytical advantage of the WAASB index. Unlike standard AMMI analysis, which often relies solely on the first principal component, WAASB integrates the variance of all significant interaction components derived from BLUP. This results in a more robust quantification of stability, as confirmed by previous studies across various crops [63–66].

In METs, WAASBY serves as a valuable simultaneous selection index, allowing breeders to weigh stability and yield according to specific breeding goals [36]. Based on this index, genotypes BOL375, BOL239, BOL376, and BOL068 were identified as superior for WSY and high stability. Similarly, genotypes BOL239, BOL375, BOL068, and BOL435 were selected for RY. Given the critical role of sugar beet in global food and energy security [14,67], identifying such resilient genotypes is essential for sustainable production under fluctuating environmental conditions [39,41,54]. In this context, WAASBY proved effective in facilitating the selection of genotypes that combine high productive potential with yield stability [41,68,69].

According to the MTSI results, genotypes with lower index values—specifically BOL434, BOL376, and BOL239—exhibited stable performance across multiple traits, indicating broad adaptability. Conversely, genotypes with higher MTSI values, such as BOL436 and BOL375, showed higher sensitivity to environmental variations, suggesting a need for breeding efforts focused on improving their resilience [70]. These findings corroborate previous studies demonstrating the effectiveness of MTSI in simultaneously selecting for yield and stability in crops like rice [40] and sugar beet [39,41].

Despite the promising identification of stable genotypes, this study has limitations. The vernalization models were parameterized using data from two cropping seasons in three locations. Validating these thresholds across a wider range of environments and long-term climatic data is necessary to confirm their global applicability. Furthermore, this study relied primarily on phenotypic stability; future research should integrate molecular validation, such as the expression analysis of bolting-related genes, to unravel the genetic mechanisms underpinning the observed phenotypic stability.

## Conclusion

This study evaluated the quantitative and qualitative performance, VT, and bolting sensitivity of sugar beet genotypes under autumn cultivation. Results indicated that while genotype BOL436 possessed the highest VT, its high sensitivity to bolting limits its stability. Conversely, genotypes BOL435 and BOL434 combined high VTs with low bolting sensitivity, rendering them resilient candidates for regions with fluctuating winter temperatures. The significant variations observed underscore the critical role of GEI in breeding programs. The WAASB, WAASBY, and MTSI indices proved effective in identifying genotypes that balance high yield with stability. Specifically, genotypes BOL375, BOL239, BOL376, and BOL068 were identified as superior for WSY stability, while BOL239, BOL375, BOL068, and BOL435 excelled in RY. These findings highlight the potential of deploying these genotypes to enhance productivity under autumn cultivation in arid regions. Future research should complement these phenotypic findings with molecular investigations into the genetic basis of stability to facilitate the development of resilient varieties. Ultimately, integrating phenotypic, genetic, and environmental data into predictive models will optimize variety selection strategies, ensuring economic viability in water-limited environments.

## Supporting information

S1 Table Daily meteorological data for experimental sites. Provides minimum and maximum temperatures for Gonbad, Dezful, and Gachsaran across two cropping years.
(XLSX)

S1 Dataset Raw experimental data. This file contains the minimal underlying data used for the predictive modeling and resilience assessment presented in this study.
(XLSX)
